# A Healthcare Edition of Sporting Equipment for Middle-Aged and Elderly

**DOI:** 10.1155/2013/745954

**Published:** 2013-12-08

**Authors:** Ching-Sung Wang, Tsung-Ching Lin, Teng-Hui Wang, Da-Lin Lee

**Affiliations:** ^1^Institute of Information and Communication Engineering, Oriental Institute of Technology, Taipei 220, Taiwan; ^2^Department of Physical Medicine and Rehabilitation, Far Eastern Memorial Hospital, Taipei 220, Taiwan; ^3^Department of Information, National Taiwan University of Science and Technology, Taipei 100, Taiwan

## Abstract

The aging phenomenon results in body organ system debilitating, which causes the balance weakening and makes a fall, fracture rate, and further medical cost to increase. The lack of exercise has been linked to increasing the incidence of hypertension, coronary artery disease, osteoporosis, degenerative arthritis, and diabetes. Chronic disease affects patients both in psychological and physiological functions which limit their daily activity. In the past, many researches pointed out that these patients can improve their balance sensation by exercise. Because of the above reasons, this research implementation forms a wireless platform of information connection system and medical data analysis. First of all, the target population in the society focuses on those elderly with the common chronic diseases, such as skeletal muscle diseases and degenerative arthritis. Using the hydraulic resist practicing equipment as the mainstay intervention can help examinee collecting the practice value and further analysis. The platform of information accords not only the data prior and after the exercise but also graphic data presentation and analysis from the medical staff members providing services in the society. It can also provide the medical unit to create data mold and a body health counselor when services in the society.

## 1. Introduction

According to the definition from WHO, it is called an aging society when the population with age greater than 65 is above 7% in the proportion. The data of population statistics in Department of Household Registration indicates the aging society of Taiwan formed in 1993. The aging rate of Taiwan is only slower than the one of Japan in Asia. How to decrease the need of long-term hospitalization and the cost in society has become an urgent issue. If the age is above 75 years' old, it is even no half of it comparing with youths [1]. Aging also affects balance system, which results in increasing risk of osteoporosis and fracture and therefore elevates medical burdens. Several researches also indicate that the balance function of those seniors is trainable, which improves their proprioception and prevents falls thereafter [2, 3].

The lack of exercise has been linked to increase in the incidence of hypertension, coronary artery disease, osteoporosis, degenerative arthritis, and diabetes. Chronic disease affects patients both in psychological and physiological functions which limit their daily activity. Aging could depress whole body functions, which also increases the risks of many chronic diseases in senior population. The study of “Senior Mental and Physical Lives in Long Term Survey” from Bureau of Health Promotion, Department of Health, Taiwan suggests that there is at least one chronic disease for 88.7% of people above 65 years and 90.9% of people above 75 years old, having three chronic diseases in 56.4%. Of those chronic diseases, the most common five are hypertension, heart or cardiovascular diseases, diabetes, osteoporosis, and arthritis. Diabetes mellitus is a chronic disorder of carbohydrate metabolism, which includes Type 1 diabetes (*β*-cell destruction, usually leading to absolute insulin deficiency), Type 2 diabetes (resulting from a progressive insulin secretory defect on the background of insulin resistance), and other specific types of diabetes and gestational diabetes mellitus. Hyperglycemia would increase the incidence of peripheral vascular diseases, sensitive nerve diseases, myocardial infarction, stroke, major arteries diseases, and other disease, which significantly increase the mortality rate of seniors.

**Figure 1:**
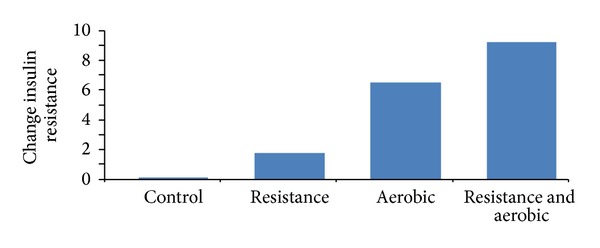
Analytics of 3 different experiment models with control group.

In 2009, Davison with his colleagues collect the data from those of 60–80 years' old and have an enlarged abdomen [4].  Figure 1 is an insulin resistance analysis test which performs three training programs: resistant, aerobic, and resistance and aerobic with one more control group. The total period of each program is 6 months with 3 times exercise per week. Of results, the combination of resistance and aerobic shows great improvement in insulin resistance. Therefore, in order to improve those physiological parameters and have better glycemic control, it is suggested for diabetic patients to process both aerobic and resistant exercises in gradual advance. Recent studies have also reported the benefits of aerobic training, resistance training, or a combination of the two on reducing glycated hemoglobin levels (HbA1c), which signifies improved glycemic control. Therefore, a healthcare of sporting equipment appeared to be needed on such exercise programs for individuals with diabetes mellitus [5–8]. Cardiovascular diseases, the ills of blood vessels (veins, arteries, and capillaries) or the heart, or both, which jeopardize the circulation of a body. The most common cardiovascular diseases are hypertension and coronary artery syndrome. Hypertension is a pressure measurement of artery which is above 140 mmHg in systolic pressure and 90 mmHg in diastolic pressure. Although the main treatment is medication, appropriate exercise would bring down blood pressure about 10–20 mmHg. However, how to detail the exercise programs has not been confirmed. Recently, it still processes the gradual resistance training program to improve seniors' blood pressure. Coronary artery atherosclerosis is the most common cause of coronary artery syndrome. It results from mal-accumulating fat in vascular wall. It results in not only angina but also in myocardial infarction, cardiac failure, or even sudden death, and so forth. Many researches also confirm that appropriate exercise could also improve cardiovascular health, which is then a benefit to the whole body. However, each exercise program still needs random test for its effect [9]. No matter it is male or female, averagely above 35 years old, the bone density would gradually decrease. The lightly or normally decreasing rate in bone density is so called osteopenia. There are many factors affecting this decreasing rate in bone density. If the density losing too fast, a disease would appear as osteoporosis, which means the bone loses its calcium resulting in honeycomb-like matrix. Since the loose skeletal system could not withstand whole body weight in his/her daily life, the fracture would therefore occur. According to the statistics, in those females about 50-year old afflicted with osteoporosis, 17.5% of them have hip fracture; 16% have vertebral fracture; and 16% also have a wrist fracture (Colles' fracture) incidence [10, 11]. The research based on the oil pressure exercise program suggests that high resistance workout could significantly improve waist and hip-neck vertebral bone density (improving values are 0.009 g/cm^2^ and 0.007 g/cm^2^ each). The following references recommend that the resistance strength and training period still need randomized tests to verify them [12–17].

**Figure 2:**
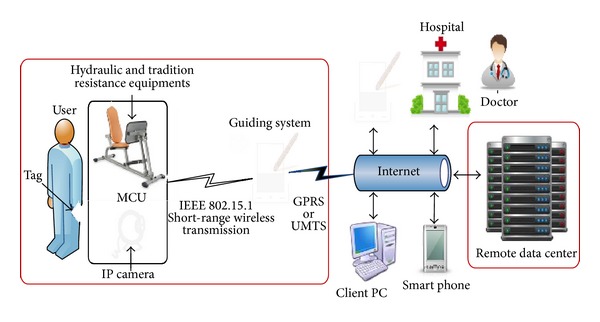
Implementation in medical data analysis platform by through the framework of cloud system.

According to Figure 2, this research suggests a system, which enables medical crews to real-timely provide their professional suggestions by through the cloud service. Adopting the characteristics of cross language and cross platform in using web services, the more resilient applications would also improve in systemic signal transportation, which therefore improves its real-time and convenient achievements.

## 2. System Description and Design

The purpose of this research is to develop an automatic platform, which further extends to the platform used to collect and analyze medical data. The developing step of this system consists of three frameworks: (1) a highly stable and convenient wireless data collecting system, (2) a mutual interaction by the function of smart phone, and (3) a remote end platform of health data managing center. The health manager website could send and share the data to this center; thus, the examinee could survey their own health information.

### 2.1. Designing a Highly Stable and Convenient Wireless Data Collecting System

#### 2.1.1. Wireless Data Collecting System

**Figure 3:**
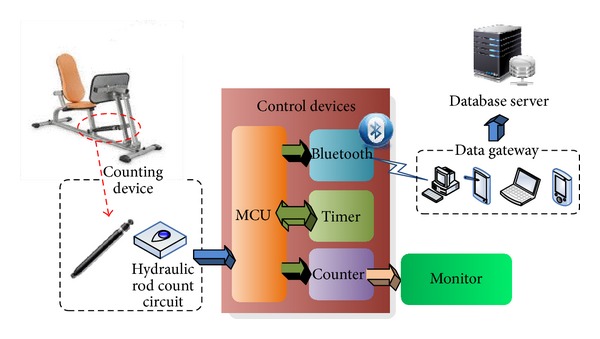
Wirelessly data collecting and transferring system.

This research uses hydraulic resistant training tool, as the major intervention equipment, and recruits 45–85 years of age, both middle-aged and elderly involved, for participation. By focusing the hydraulic rod on the training equipment, this research edits an installable counting device for every hydraulic exercise device. By maintaining the original shape of exercise equipment as the prerequisite, that hangs the counting devise on the hydraulic rod of exercise equipment improves the repeating use rate and is substitutable. In order to further improve its convenience, this research equips an additional sensor of sliding on the system for counting the number of times the user uses the equipment as shown in Figure 3.

The control device is set up for signal receiving, timing, wirelessly data transporting, and numbers presenting. When user operates first pull on the hydraulic rod of the system, the sensor of sliding would signal to the control device. Based on the time set, it counts back when the training equipment is used and alarms out and sums up a total at final. Through the Bluetooth transportation, it transfers this data to the monitor and presents the number of operating on the monitor. Monitor mainly consists of Bluetooth communication function, which thus improves systemic integrity, greatly minimizes the quantity of devices, directly receives exercise records from control devices, and finally uploads data to the cloud inventory.

#### 2.1.2. Identification System

As inFigure 4, to identify the user, a device of IP Camera and two-dimension unit code achieves is used. Every time before an exercise, ID card with printed QR code, screened in front of IP Camera and decoded to command a microcontroller, triggers the counting device. When the time is over, the system will automatically record the data and the period of exercise.

### 2.2. Mutual Interaction of Smart Phone

**Figure 5:**
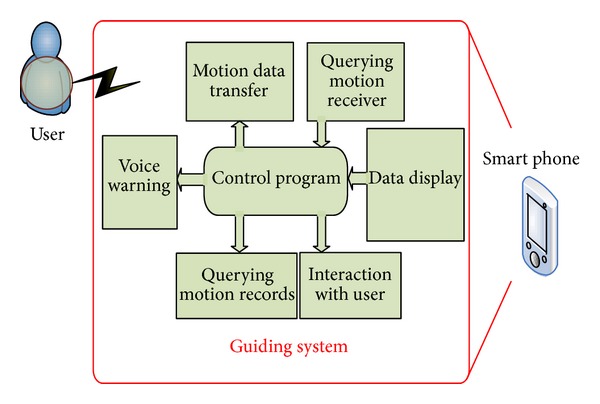
Smart phone mutual commutable function and management program.

Combining interactive dictation function within application function list of smart phone and then adopting multitouch panel and intrinsic Bluetooth and phonetic functions, and so forth, the dictations are achieved by audio alarm and suggestion, which enables users not only to avoid sport injury but also to simulate actual personnel dictation, to record training history, and to save in intrinsic memory of the smart phone. Finally, it appears as shown in Figure 5, presenting appropriate selection bottoms and suitable accessary instructions to prevent the error exercise causing sport injury.

### 2.3. Health Information Manager Platform at the Data Center of Remote End

This research develops a versatile and interactive web interface. Based on the medical doctor's suggestion and cooperation in one-month period data collection, this system adopts models website framework with service-oriented architecture and web operating system. Concerning the operating convenience and the website accommodation, the website, through the wireless pathway, would transfer the physiologic data to this system, which not only decreases systemic repetition but also provides versatile interfaces for different authorities.

## 3. Data Management Center and Health Service Platform

**Figure 6:**
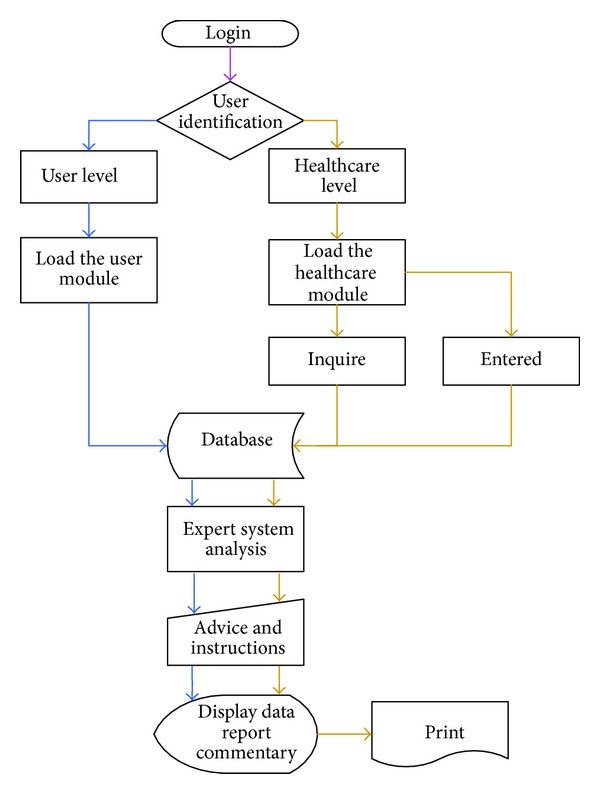
Medical information analysis platform for system workflow.

(1) The individual health management function provides the user with the independent health management service. Once the user completes the health checkup, the healthcare personnel will input the checkup data into the cloud database via the Internet. The web interface allows the users and their families to query the users' health checkup results and related data via mobile devices or the PCs at any time without installing any other tool [18].

(2) The system distributes the online users to the specific level based on the privilege of the account; thus, the users will be distributed to the user level and linked with the database of the checkup data for data access and analysis. The expert system will display the analyzed checkup data together with the brief interpretation for the results on the webpage. For example, systolic pressure is higher than 120; diastolic pressure is higher than 80; heartbeat is higher than 100/min or lower than 60/min [19]. The system will display too higher/lower blood pressure and too higher/lower heartbeat and export the graphic analysis report for reference of the users and healthcare personnel. When the healthcare personnel use the system, the system will distribute them to the healthcare level and then load the healthcare module, providing them with the functions of querying, printing, and inputting as shown in Figure 6.

(3) The healthcare personnel input the physical checkup records into the database via the web browser and the inputting application provided by WebOS. After that, the expert system will process the data and save them to the right location of the database. As for the service of medical information analysis platform, it allows the users, their families, and the medical care institutions to obtain the status of the users' health checkup through the devices, such as PC or smart device, which also provides the analysis reports and brief interpretation of the checkup results. The operation interface of the platform adopts the modular web architecture. Besides reducing the repeated system design, it could also provide different functional interfaces based on different privileges. Below is the introduction of the functions provided by the health management service platform built in the system.

## 4. Implementation Results

**Figure 7:**
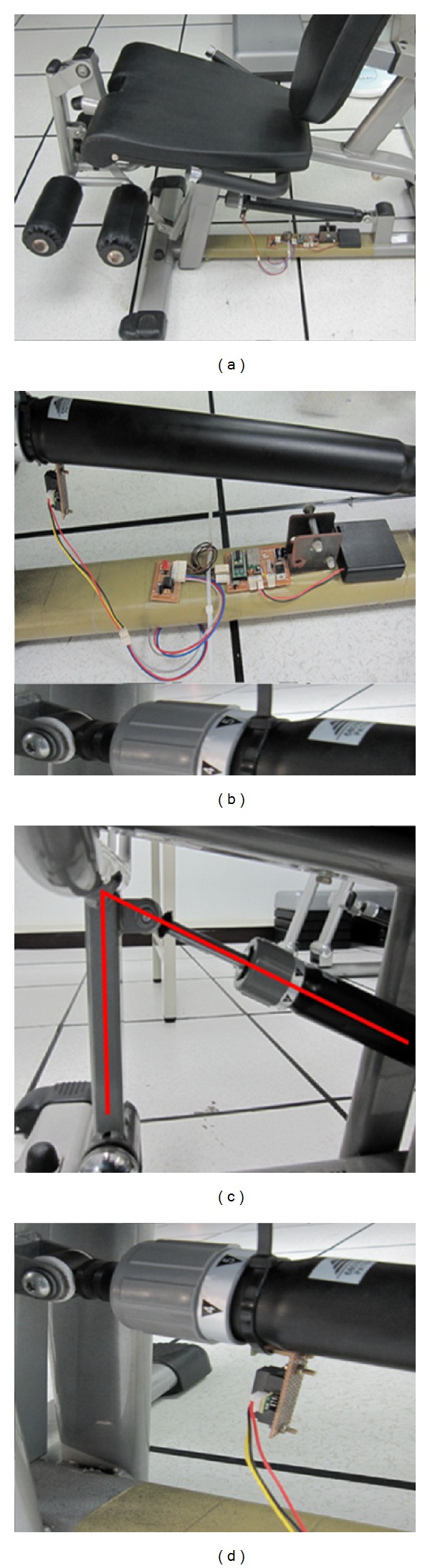
Image of detecting device set on pressured sport equipment.

This system hangs counting device on a hydraulic rod in order to improve the utilization, update, and replacement rate.  Figure 7 is shown to set up a training machine which consists of infrared, microcontroller, power supply, and circuit, exemplifying the system's extensions in (1) chest pushing, (2) thigh adducting and abducting, (3) arm lifting, (4) leg straightening, (5) back scratching with hand rising; (6) knee extending are able to achieve great stability and practicality.

**Figure 8:**
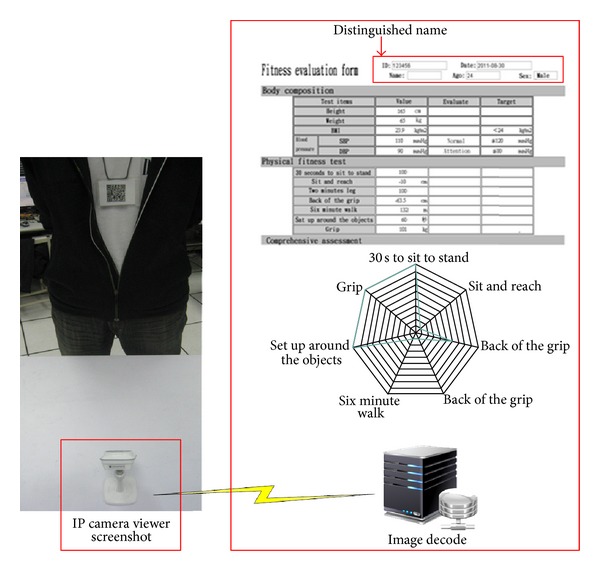
Data analysis report.

The physical data analysis function stores and analyzes the physical checkup data in the database as shown in Figure 8. The users' checkup data are stored monthly and monitored at the same time. When the value is beyond the range of the physician's advice or the system settings, it will display the abnormal data of the case on the interface, analyze the reports graphically, and provide brief interpretation of the checkup results, which could help the physicians to learn the physical status of the patients.

## 5. Conclusion

Based on the research, hydraulic resistant exercise can be recommended for those with osteoporosis for improving bone density. It also responses to the government advocating whole citizen sport. In addition, using the hydraulic resist practicing equipment as the mainstay intervention can help examinee collecting the practice value and further analysis. The platform of information not only accords these values into the data prior and after the exercise, but also helps providing graphic data presentation and analysis from the medical staff members providing services in the society. It can also provide the medical unit to create data mold and a body health counselor when services in the society.

## Figures and Tables

**Figure 4:**
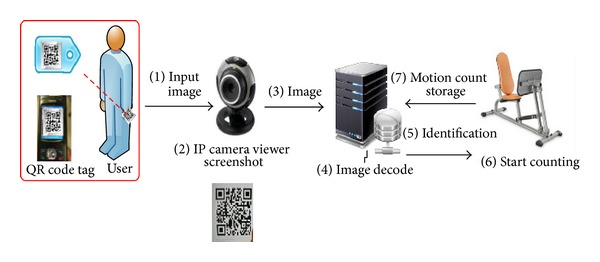
User's identification system.
